# Decrements in health‐related quality of life associated with adverse events in people with diabetes

**DOI:** 10.1111/dom.14610

**Published:** 2021-12-20

**Authors:** Mi Jun Keng, Jose Leal, Louise Bowman, Jane Armitage, Borislava Mihaylova, ASCEND Study Collaborative Group

**Affiliations:** ^1^ Health Economics Research Centre, Nuffield Department of Population Health University of Oxford Oxford UK; ^2^ British Heart Foundation Centre of Research Excellence Oxford UK; ^3^ Clinical Trial Service Unit and Epidemiological Studies Unit, Nuffield Department of Population Health University of Oxford Oxford UK; ^4^ Medical Research Council Population Health Research Unit, Nuffield Department of Population Health University of Oxford Oxford UK; ^5^ Wolfson Institute of Population Health Queen Mary University of London London UK

**Keywords:** cardiovascular disease, diabetes complications, health economics

## Abstract

**Aim:**

To estimate the decrements in health‐related quality of life (QoL) associated with a range of adverse events to inform assessments of the effects of diabetes treatments on QoL in contemporary clinical practice.

**Methods:**

Participants' QoL utility measures were derived from the five‐level EuroQoL five‐dimensional (EQ‐5D‐5L) questionnaires completed by 11 683 ASCEND participants (76% of 15 480 recruited). EQ‐5D utility decrements associated with cardiovascular (myocardial infarction, coronary revascularization, transient ischaemic attack [TIA], ischaemic stroke, heart failure), bleeding (gastrointestinal [GI] bleed, intracranial haemorrhage, other major bleed), cancer (GI tract cancer, non‐GI tract cancer), and microvascular events (end‐stage renal disease [ESRD], amputation) were estimated using a linear regression model following adjustment for participants' sociodemographic and clinical risk factors.

**Results:**

Amputation was associated with the largest EQ‐5D utility decrement (−0.206), followed by heart failure (−0.185), intracranial haemorrhage (−0.164), GI bleed (−0.091), other major bleed (−0.096), ischaemic stroke (−0.061), TIA (−0.057), and non‐GI tract cancer (−0.026). We were unable to detect decrements in EQ‐5D utility associated with myocardial infarction, coronary revascularization, GI tract cancer, or ESRD. EQ‐5D utility was lower at older age, independent of other factors.

**Conclusion:**

These estimated decrements in QoL associated with cardiovascular, bleeding, cancer, and other adverse events can inform assessments of the overall value of treatments in patients with diabetes.

## 1 INTRODUCTION

People with diabetes are at an increased risk of a range of adverse events related to the disease and co‐morbidities that develop over the lifetime.^1^, ^2^ Diabetes interventions typically need to be evaluated over the lifetime to fully capture their impacts. Additionally, some treatments may pose safety concerns^3^, ^4^ and the trade‐offs between benefits and risks need to be assessed. This is challenging when it is difficult to compare the severity of different adverse events.

Decision‐analytic models are frequently used for evaluating diabetes interventions. These models can combine the impacts of treatment across a range of outcomes to assess net health effects and/or cost‐effectiveness over the long term.^5^, ^6^ Quality‐adjusted life‐years (QALYs), a combined measure of life expectancy and health‐related quality of life (QoL), is a widely adopted metric used to quantify the effectiveness of interventions.^7^ In the UK, the National Institute of Health and Care Excellence (NICE) requires health effects to be expressed in terms of QALYs for health technology assessments.^8^ Thus, decrements in QoL associated with adverse events are needed to inform such models.

Estimates of decrements in QoL associated with common cardiovascular and microvascular complications in diabetes have been reported previously.^9^, ^10^, ^11^, ^12^, ^13^, ^14^, ^15^, ^16^, ^17^, ^18^ The last study that reported estimates for a UK‐based cohort was the United Kingdom Prospective Diabetes Study (UKPDS), with QoL surveys conducted from 1996 to 2007.^11^ These results may be outdated given improvements in management and treatments of diseases since then. Estimates from more recent multinational studies that include participants from the UK may not truly reflect the preferences of patients in the UK because of regional variations in how people report their health status.^19^ Using data from the recent ASCEND study,^20^ we provide updated results for the UK context, as well as first estimates for bleeding and cancer events in people with diabetes.

## 2 METHODS

### 2.1 Measurement of health‐related QoL in the ASCEND study

Details of the ASCEND study have been reported previously.^20^, ^21^, ^22^ Briefly, ASCEND was a 2 × 2 factorial design trial that randomized 15 480 participants with established diabetes but without previous cardiovascular disease to 100 mg aspirin daily or matching placebo, and separately, to a 1‐g capsule containing omega‐3 fatty acids daily or matching placebo. Participants were recruited from 2005 to 2011, and followed for an average of 7.4 years until 2017. QoL for ASCEND participants was measured by utility scores derived from the five‐level version of the EuroQoL five‐dimensional (EQ‐5D‐5L) questionnaire.^23^ The EQ‐5D‐5L questionnaire was mailed to participants towards the end of follow‐up with responses collected during 2016 and 2017, an average of 6.7 years into study follow‐up. This excluded participants who were lost to follow‐up, participants followed up through administrative data only, or participants who had died. Participants were asked to rate their health across five domains—namely, mobility, self‐care, usual activities, anxiety/depression, and pain—by selecting one of five possible levels: ‘no problem’, ‘slight problems’, ‘moderate problems’, ‘severe problems’, or ‘extreme problems/unable to’. These responses were mapped onto EQ‐5D utility values, with 1 representing perfect health, 0 representing health state equivalent to death, and values less than 0 representing a health state worse than death. In line with NICE's latest position statement,^24^ the EQ‐5D utility values were calculated by mapping onto the UK EQ‐5D‐3L value set using the mapping function developed by van Hout et al.^25^ (van Hout tariff). Alternative tariff values, developed by Hernandez Alava et al.^26^ for the NICE Decision Support Unit (DSU tariff), were used in sensitivity analysis.

### 2.2 Identifying adverse events

The adverse events of interest included: (a) cardiovascular disease: myocardial infarction, urgent and non‐urgent coronary revascularization, transient ischaemic attack (TIA), ischaemic stroke, heart failure; (b) bleeding: intracranial haemorrhage, gastrointestinal (GI) bleed, other major bleed (excluding eye bleed); (c) cancer: GI tract cancer and non‐GI tract cancer (excluding non‐melanoma skin cancer); and (d) other diabetes complications: amputation (lower limb) and end‐stage renal disease (ESRD). These events were identified using adverse events reported within the ASCEND study and linked routine hospital admissions data. Further details are provided in Table S1.

### 2.3 Statistical methods

The EQ‐5D utility data are bounded above by 1 and positively skewed, with a high proportion of participants reporting full health (utility value of 1). Hence, for modelling the EQ‐5D utility decrements associated with adverse events, in addition to the frequently used ordinary least squares (OLS) regression, generalized linear models (GLMs), Tobit models, beta regression models, and two‐part models (logistic equation for the first part, GLMs for the second part), were also considered. The selection of the most appropriate model was based on predictive performance and parsimony (further details can be found in Appendix **S1**).

Candidate covariates of the statistical model were: time since occurrence of adverse events as a categorical variable (no history, event occurred within 1 year prior to questionnaire response, and event occurred more than 1 year prior to questionnaire response), age and duration of diabetes at time of questionnaire response, sociodemographic characteristics and clinical risk factors measured at recruitment into the trial (Table S2). The time of adverse event occurrence was split into three levels to reflect the acute and longer term impact on EQ‐5D utility associated with the adverse event occurrence.^11^, ^17^, ^18^, ^27^ These levels were combined if there were fewer than 10 events in each level or if there was no evidence of difference between acute and longer term decrements in EQ‐5D utility based on the Wald test (*P* ≥ .05). Natural cubic splines were fitted to test for and explore the shape of non‐linearity of age. Stepwise selection of covariates was performed based on the likelihood ratio test, with *P* less than .1 and *P* less than .05 used for inclusion and exclusion, respectively.

Missing categorical patient characteristics were imputed with the modal category value. Missing clinical risk factors were imputed by predictive mean matching using the method of multivariate imputation by chained equations, with all covariates and history of event occurrence at the time of EQ‐5D questionnaire response included in the imputation model. Missing questionnaire responses were imputed at the domain level, using the same approach, with the visual analogue scale score further included in the imputation model.

## 3 RESULTS

**FIGURE 1:**
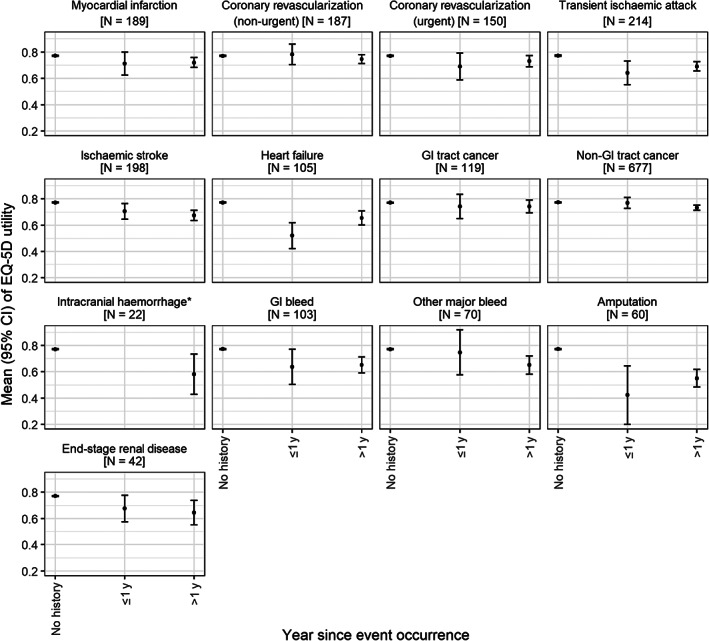
EuroQoL five‐dimensional (EQ‐5D) utility by adverse event and time since event occurrence. *For intracranial haemorrhage, there were too few participants who experienced an event within 1 year prior to EQ‐5D response, so the EQ‐5D utility was presented for participants who had experienced an event irrespective of time of event. ≤1 y, an event occurred within 1 year prior to EQ‐5D questionnaire response; >1 y, an event occurred more than 1 year prior to EQ‐5D questionnaire response; GI, gastrointestinal. Other major bleed refers to bleeding events that are neither intracranial haemorrhage nor GI bleed. The number in brackets is the number of participants who had experienced a particular adverse event by the time of the EQ‐5D questionnaire

At the time the EQ‐5D questionnaire was mailed, of the 15 480 participants in ASCEND, 2305 (14.9%) were lost to follow‐up or followed up through administrative data only, and 1492 (9.6%) had died. The characteristics of the remaining 11 683 participants included in the analysis are presented in Table 1 (see Table S3 for characteristics after imputation). Ninety‐four percent of participants included in the analysis had type 2 diabetes. At the time of EQ‐5D questionnaire response, participants' mean age was 68 years and their mean duration of diabetes was 16 years. Eleven thousand two hundred and forty‐seven (96.3%) participants responded to the questionnaire, with 11 028 (98.1%) responding to all five domains (Table S4). About 60% of participants reported some pain/discomfort, 40% reported some problems with mobility and performing usual activities, 30% reported some problems with depression/anxiety, and 15% reported some problems with self‐care (Table 2). The mean EQ‐5D utility across the cohort was 0.773, with 3110 (27%) participants reporting full health (Table S5). The mean EQ‐5D utility for participants who did not experience an event was 0.781, decreasing to 0.722 and 0.683 for participants who had experienced one or two adverse events, respectively (Table S6). The adverse event that participants had experienced the most by the time of EQ‐5D questionnaire response was non‐GI tract cancer (661 [5.7%]), followed by transient ischaemic attack (214 [1.8%]), ischaemic stroke (198 [1.7%]), myocardial infarction (189 [1.6%]), non‐urgent coronary revascularization (187 [1.6%]), urgent coronary revascularization (150 [1.3%]), GI tract cancer (146 [1.3%]), heart failure (105 [0.9%]), GI bleed (103 [0.8%]), other major bleed (70 [0.6%]), amputation (60 [0.5%]), ESRD (42 [0.4%]), and intracranial haemorrhage (22 [0.2%]). The unadjusted mean EQ‐5D utility stratified by time since occurrence of each adverse event is summarized in Figure 1 and Table S7.

**TABLE 1:** Characteristics of 11 683 participants included in the analysis

	N (%) or mean (SD)
Participant characteristics at entry into the ASCEND study	
Diabetes type	
1	707 (6.1%)
2	10 976 (93.9%)
Sex	
Male	7347 (62.9%)
Female	4336 (37.1%)
Smoking status	
Current smoker	813 (7.0%)
Former/never smoker	10 734 (91.9%)
Missing	136 (1.2%)
Race	
White	11 282 (96.6%)
Indian/Pakistani/Bangladeshi	147 (1.3%)
African/Caribbean	86 (0.7%)
Missing	168 (1.4%)
Townsend index^a^	
Q1: <−2.42 (least deprived)	5232 (44.8%)
Q2: ≥−2.42, <−0.44	2934 (25.1%)
Q3: ≥−0.44, <1.79	1851 (15.8%)
Q4: ≥1.79, <4.75	1237 (10.6%)
Q5: ≥4.75 (most deprived)	403 (3.4%)
Missing	26 (0.2%)
Hypertension	
Y	7173 (61.4%)
N	4435 (38.0%)
Missing	75 (0.6%)
Diabetic retinopathy	
Y	2232 (19.1%)
N	9359 (80.1%)
Missing	92 (0.8%)
Use of statin	
Y	8919 (76.3%)
N	2764 (23.7%)
Use of ACE inhibitor or ARB	
Y	6818 (58.4%)
N	4865 (41.6%)
Age (y)	62.8 (8.5)
Diabetes duration (y)	9.7 (9.3)
Missing	588 (5%)
Body mass index (kg/m^2^)	31.0 (6.4)
Missing	82 (0.7%)
HbA1c (IFCC mmol/mol)	54.4 (12.4)
Missing	4165 (35.7%)
HDL cholesterol (mmol/L)	1.27 (0.35)
Missing	4175 (35.7%)
Non‐HDL cholesterol (mmol/L)	2.88 (0.83)
Missing	4175 (35.7%)
Systolic blood pressure (mmHg)	135.9 (15.0)
Missing	3192 (27.3%)
Diastolic blood pressure (mmHg)	77.3 (9.3)
Missing	3197 (27.4%)
Urinary albumin/creatinine ratio (mg/mmol)^b^	
<3	6644 (56.9%)
≥3	854 (7.3%)
Missing	4185 (35.8%)
eGFR (mL/min/1.73m^2^)^c^	
<45	209 (1.8%)
≥45, <60	584 (5.0%)
≥60, <90	3081 (26.4%)
≥90	3645 (31.2%)
Missing	4164 (35.6%)
Participant characteristics at EQ‐5D questionnaire response	
Age (y)	68.5 (8.3)
Diabetes duration (y)	16.4 (9.4)

*Note*: Only 7589 (65%) participants returned usable blood/urine sample in the study.

Abbreviations: ACE, angiotensin‐converting enzyme; ARB, angiotensin II receptor blocker; eGFR, estimated glomerular filtration rate; HDL, high‐density lipoprotein; IFCC, International Federation of Clinical Chemistry.

^a^ Townsend index stratified according to range of scores of 2011 UK population quintiles.

^b^ 1896 (25%) participants who had undetectable albumin levels were reclassified as having no albuminuria (urinary albumin/creatinine ratio < 3).

^c^ Calculated from blood cystatin C concentration using the CKD‐EPI formula.

**TABLE 2:** EuroQoL five‐dimensional questionnaire responses from 11 683 participants

	Mobility	Self‐care	Usual activities	Pain/discomfort	Anxiety/depression
1 No problem	6212 (53.2%)	9494 (81.3%)	6868 (58.8%)	4204 (36.0%)	7905 (67.7%)
2 Slight problems	2453 (21.0%)	970 (8.3%)	2264 (19.4%)	4041 (34.6%)	2133 (18.3%)
3 Moderate problems	1641 (14.0%)	545 (4.7%)	1430 (12.2%)	2053 (17.6%)	923 (7.9%)
4 Severe problems	836 (7.2%)	137 (1.2%)	492 (4.2%)	768 (6.6%)	144 (1.2%)
5 Extreme problems/unable to	39 (0.3%)	16 (0.1%)	119 (1.0%)	111 (1.0%)	47 (0.4%)
Missing	502 (4.3%)	521 (4.5%)	510 (4.4%)	506 (4.3%)	531 (4.5%)

*Note*: Summary values are N (%).

### 3.1 Decrements in EQ‐5D utility associated with adverse events

**FIGURE 2:**
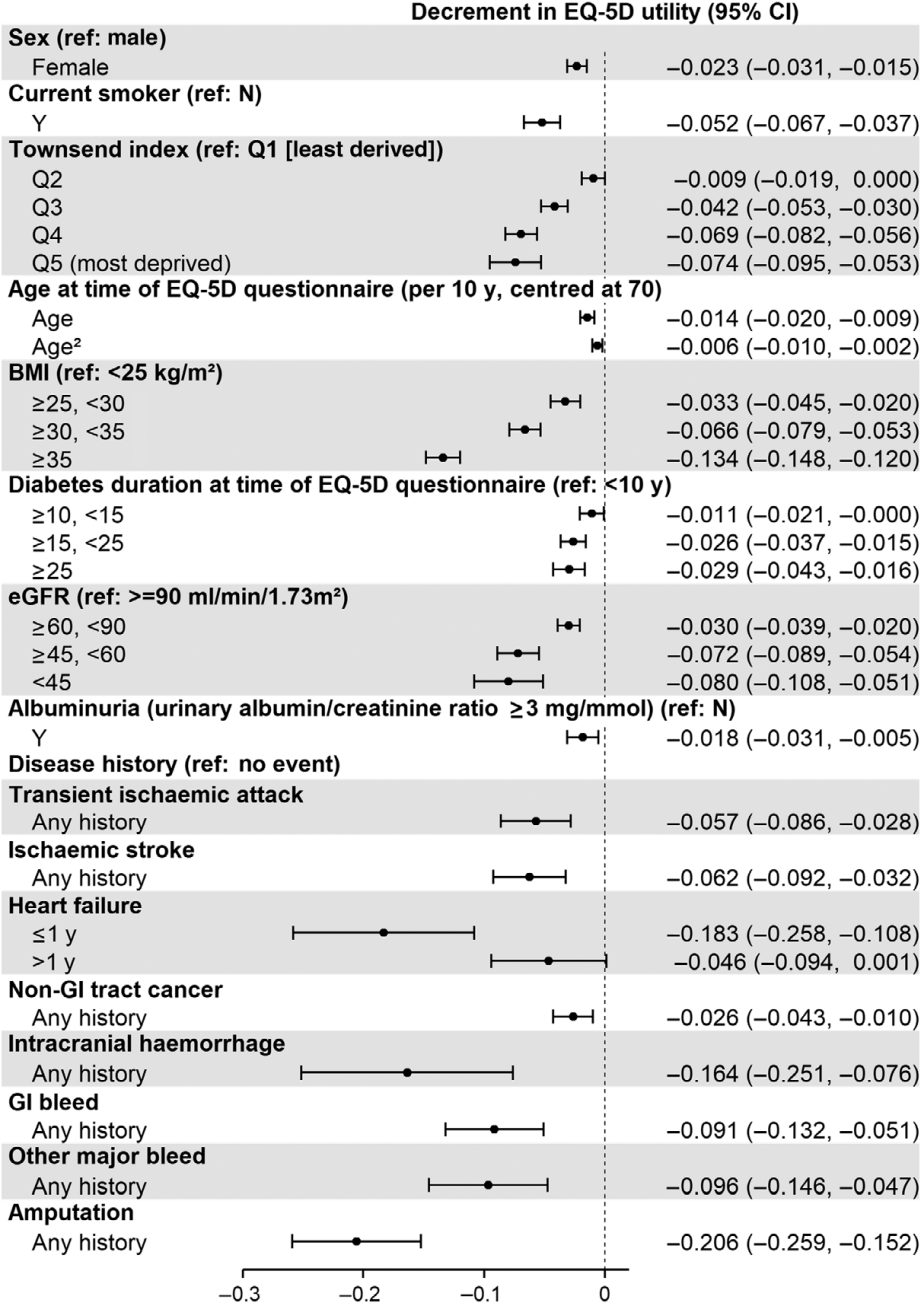
EuroQoL five‐dimensional (EQ‐5D) utility in people with diabetes associated with patient characteristics, clinical factors, and adverse events. The EQ‐5D utility for the reference individual (male, not current smoker, living in least deprived region, aged 70, BMI < 25 kg/m^2^, diabetes duration < 10 years, eGFR ≥ 90 mL/min/1.73m^2^, no albuminuria, with no disease history) is 0.906 (0.891, 0.920). ≤1 y, an event occurred within 1 year prior to EQ‐5D questionnaire response; >1 y, an event occurred more than 1 year prior to EQ‐5D questionnaire response; BMI, body mass index; eGFR, estimated glomerular filtration rate; GI, gastrointestinal. Other major bleed refers to bleeding events that are neither intracranial haemorrhage nor GI bleed. We were unable to detect decrements in EQ‐5D utility associated with myocardial infarction, coronary revascularizations, GI tract cancer, and end‐stage renal disease, so these events were not included in the model

The OLS model performed at least as well as other candidate models and was chosen for parsimony (see Appendix **S1**). The mean EQ‐5D utility value for a 70‐year‐old male who is not a current smoker, living in the least deprived region, with body mass index (BMI) less than 25 kg/m^2^, diabetes duration of less than 10 years, an estimated glomerular filtration rate (eGFR) of 90 mL/min/1.73m^2^ or higher, no albuminuria, and no disease history, was 0.906 (95% CI, 0.891, 0.920) (Table S8). Relative to this reference case, the decrements in EQ‐5D utility associated with sociodemographic and clinical risk factors, and the occurrence of adverse events, are presented in Figure 2.

Other than heart failure, there was no evidence of differential impacts of adverse events on EQ‐5D utility between acute phase (in year of event) and longer term (in later years). Overall, amputation was associated with the largest EQ‐5D utility decrement (−0.206 [−0.259, −0.152]). For cardiovascular events, heart failure was associated with the largest EQ‐5D utility decrement in the year of event (−0.183 [−0.258, −0.108]), but in subsequent years, its effect was smaller and not statistically significant (−0.046 [0.094, 0.001]). TIA (−0.057 [−0.086, −0.028]) and ischaemic stroke (−0.062 [−0.092, −0.032]) were associated with similar decrements in EQ‐5D utility. Among bleeding‐related events, intracranial haemorrhage was associated with the largest EQ‐5D utility decrement (−0.164 [−0.251, −0.076]), followed by GI bleed (−0.091 [−0.132, −0.051]) and other major bleed (−0.096 [−0.146, −0.047]). Non‐GI tract cancer was also associated with an EQ‐5D utility decrement (−0.026 [−0.043, −0.010]). We were unable to detect decrements in EQ‐5D utility associated with myocardial infarction, coronary revascularizations, GI tract cancer, or ESRD, so these events were not included in the final model.

### 3.2 Decrements in EQ‐5D utility associated with other factors

Independent of the occurrence of adverse events, being female, a smoker, living in a more deprived region, having higher BMI, a longer duration of diabetes, and decreased renal function, were all associated with lower EQ‐5D utility. In particular, morbid obesity (BMI ≥ 35 kg/m^2^) was associated with lower EQ‐5D utility (−0.134 [−0.148, −0.120]) relative to normal weight (BMI < 25 kg/m^2^). We also observed a generally lower EQ‐5D utility with increasing age. EQ‐5D utility remained comparatively stable below 70 years of age (difference <0.010 from 50 to 70 years old), but then decreased beyond 70 years of age (Figure **S1**). Following adjustments for other covariates, relative to a 70‐year‐old, the EQ‐5D utility of an 80‐ and a 90‐year‐old was lower by −0.020 and −0.053, respectively.

Results were similar in the sensitivity analysis in which the DSU tariff was used to calculate the EQ‐5D utility values instead of the van Hout tariff (Table S9).

## 4 DISCUSSION

In this study, we estimated the decrements in EQ‐5D utility associated with cardiovascular, bleeding, cancer, and other diabetes‐related events among the 11 683 participants in the ASCEND study. We found that amputation was associated with the largest EQ‐5D utility decrement (−0.206). This was followed by heart failure (−0.185), intracranial haemorrhage (−0.164), GI bleed (−0.091), other major bleed (−0.096), ischaemic stroke (−0.061), TIA (−0.057), and non‐GI tract cancer (−0.026). Following adjustments for participants' sociodemographic and clinical risk factors and the occurrence of other adverse events, we were unable to detect decrements in EQ‐5D utility associated with myocardial infarction, coronary revascularizations, GI tract cancer, and ESRD.

The EQ‐5D‐5L responses from ASCEND participants (mean age 68 years at time of EQ‐5D‐5L response) were largely in line with EQ‐5D‐5L responses from adults aged 65‐74 years in the 2018 Health Survey for England (HSE).^28^ Pain/discomfort was the most commonly reported problem, with about 60% of participants reporting at least some problems in both surveys. This was followed by problems with mobility and usual activities, although these problems were more prevalent among ASCEND participants (for mobility, 42% in ASCEND vs. 33% in HSE; for usual activities, 37% in ASCEND vs. 25% in HSE). At least some problems with depression/anxiety and, separately, self‐care, were reported by about 30% and 15% of participants in both surveys.

Decrements in QoL associated with diabetes‐related complications have been reported in several previous studies.^9^, ^10^, ^11^, ^12^, ^13^, ^14^, ^15^, ^16^, ^17^, ^18^ The estimated decrements in QoL associated with myocardial infarction, stroke, and heart failure were somewhat smaller in more recent studies (Table S10). For example, stroke was associated with −0.046 and −0.099 lower QoL in the LEADER^16^ and ADVANCE^13^ studies, respectively, in contrast to the larger −0.165 decrement in the older UKPDS study.^11^ This may be a reflection of improvements in treatment and management of cardiovascular disease over the years. Our study, which uses the latest QoL data, found a generally similar impact of heart failure on QoL to other recent studies. Our study is the first to differentiate the QoL decrement associated with ischaemic and haemorrhagic stroke. We found intracranial haemorrhage to have more than twice the impact on QoL than ischaemic stroke. Given that more than 80% of strokes are ischaemic strokes,^29^ the combined QoL decrement of stroke in our study would be roughly similar to estimates from other more recent studies. We found no evidence of an acute impact of myocardial infarction on QoL that has been observed in other studies, which could be because of the decreasing impact over time (a QoL decrement of −0.028 was reported in the ACCORD and Look AHEAD studies^18^ in contrast to −0.065 in the UKPDS). In addition, there were only 34 myocardial infarction events in the year prior to the EQ‐5D questionnaire response, which may be too small a number to detect a small effect. There was also no evidence of differential impacts on QoL between the acute and longer term periods following stroke and amputation, as observed in the ACCORD and Look AHEAD studies, possibly also because of the small number of events (42 ischaemic stroke events, 10 amputation events in the year prior to EQ‐5D questionnaire response). We did observe a separate acute impact of heart failure (−0.183), although this was much larger in magnitude than observed in the ACCORD and Look AHEAD studies (−0.051). It should be noted that the ACCORD and Look AHEAD studies used the Health Utilities Index Mark 3 (HUI‐3) instrument to measure QoL, which may limit comparability. The finding that amputation is associated with the largest QoL reduction is consistent with observations in other studies,^10^, ^11^, ^12^, ^13^ although the QoL decrement we observed is considerably greater than those reported in other studies. In the model that included all disease histories (Table S9), we found a similar magnitude of QoL decrement associated with ESRD (−0.036 [−0.102, 0.029]) to previous studies.^13^, ^18^ However, this was not statistically significant, again possibly because there were only 42 people with ESRD and a QoL measurement in ASCEND. The ESRD effect, therefore, may have not been reliably captured.

There are a limited number of studies reporting decrements in QoL associated with bleeding events,^30^, ^31^, ^32^ and none specific to people with diabetes. The QoL decrements associated with bleeding events in ASCEND (GI bleed −0.092, other major bleed −0.096) were larger than those reported in other studies (e.g. in ENGAGE AF‐TIMI 48,^32^ −0.029 at 3 months after GI bleed, −0.029 at 3 months after other major extracranial bleed; in TRANSLATE‐ACS,^30^ −0.0391 after bleeding academic research consortium type 2‐4 bleed) (Table S11). Participants in both these studies had previous cardiovascular disease, so the additional change in QoL from a bleeding event on top of their existing cardiovascular disease could be lower than in people who did not previously have cardiovascular disease, as in ASCEND. We were unable to explore the interactions between bleeding and cardiovascular disease as there were fewer than 10 participants who had both bleeding and cardiovascular events prior to the EQ‐5D questionnaire.

We observe an association between obesity and lower QoL, independent of co‐morbid conditions. In particular, the QoL decrement from being morbidly obese relative to being of normal weight is even more pronounced than experiencing an ischaemic stroke. This is concordant with previous reports,^10^, ^14^, ^33^ with findings suggesting that obesity in itself may impair physical functioning and cause health distress, leading to lower QoL, irrespective of obesity‐related co‐morbidities.

The QoL decline with older age observed in this study, in addition to the occurrence of disease events, was previously reported in the ADVANCE study.^13^ However, the impact we observe is much smaller. For example, compared with a patient aged 70 years, a patient aged 80 years (all other factors being the same) has 0.03 lower QoL (inclusive of the effect of increasing duration of diabetes) in contrast to 0.08 lower QoL reported in ADVANCE. This could be a reflection of the generally healthier patient population in ASCEND, which recruited people without previous cardiovascular disease, in contrast to ADVANCE, where 32% of participants had cardiovascular disease at entry.

In addition to temporal changes in QoL impacts from improvements in healthcare, differences in population characteristics, and differences in the instruments used to measure QoL that have been described above, another key factor that could contribute to differences in estimates observed in ASCEND and in other studies is the methodology used. Studies with repeated measures of QoL could control for unobserved variation of QoL across participants (e.g. people with lower QoL independent of modelled characteristics and disease history experiencing an adverse event) by using fixed effects models, for example. This typically results in smaller estimates of QoL decrements associated with adverse events.^11^, ^13^ We had only one measure of QoL per participant in ASCEND, which could be a reason for the larger estimated QoL decrements associated with amputation and bleeding events than those reported in other studies.^13^, ^18^, ^30^, ^32^ We acknowledge this as a limitation of our study.

Another limitation of our study is that participants who died prior to QoL measurement could not contribute to the study, so there may be survival bias. In applications, QoL is evaluated concurrently with survival in assessing the net effects of treatments, and our analysis is in line with how these estimates are intended for use. This does make the assumption that the QoL decrements estimated also apply to those participants who have since died, while they were still alive. This may not be true if, for example, participants who survived have experienced a better recovery from adverse events, and thus may rate their health more positively than participants who experienced an event and later died. Second, there may be volunteer bias where healthier or more health‐conscious people were enrolled into the study, and these people may adhere to medical treatments better than the average person with diabetes. This is a common problem across QoL studies using clinical trial data, and we acknowledge that it is difficult to quantify the impact this has on our results. Lastly, we were not able to investigate interactions between different complications because of small numbers of co‐occurring events. The most frequent co‐occurring events in ASCEND were myocardial infarction and urgent coronary revascularizations (N = 140), and stroke and TIA (N = 44). We did not detect any significant interactions between these events. There were fewer than 20 co‐occurrences of all other event combinations. Previous studies have not found significant interactions between events, suggesting that the additive specification of the effects of experiencing multiple conditions is justified.^11^, ^13^

Despite the limitations above, our study benefits from the high quality of data in ASCEND. Other than heart failure, amputation, and ESRD, all other endpoints were clinically adjudicated. This made it possible, for example, to categorize strokes, which are usually suboptimally recorded in routinely collected health data.^34^ There was also good completeness of EQ‐5D‐5L response, with 94% of participants included in the analysis responding to all five domains of the EQ‐5D questionnaire. Additionally, ASCEND consisted exclusively of participants from the UK. This mitigates the issue of regional variations in how people report their health status that have been reported previously,^19^ which may affect estimates from multinational studies like LEADER, SAVOR‐TIMI 53, and ADVANCE. Our study provides the latest estimates of QoL decrements associated with a wide range of adverse events in patients with diabetes, particularly for the UK context, where the last study was the UKPDS with QoL surveys conducted from 1996 to 2007. Our study is also the first to report separate estimates for ischaemic stroke and intracranial haemorrhage, as well as estimates for other bleeding events for people with diabetes. These estimates will be useful, for example, in evaluating net benefits of antiplatelet and anticoagulant treatments in people with diabetes where cardiovascular benefits need to be weighed against bleeding risks.^35^, ^36^, ^37^

In conclusion, our study provides contemporary estimates of decrements in QoL associated with a wide range of cardiovascular, bleeding, cancer, and microvascular events in people with diabetes. These estimates will be useful to inform the effectiveness and cost‐effectiveness of diabetes treatments.

## CONFLICT OF INTEREST

All authors declare no conflict of interest.

## AUTHOR CONTRIBUTIONS

MJK, JL, and BM designed the study. MJK conducted the analyses and drafted the manuscript. JA and LB are principal investigators of the ASCEND study and provided important clinical insights in the development of the study and interpretation of results. All authors interpreted the results, critically revised and approved the final version of the manuscript. MJK is the guarantor of this work and, as such, had full access to all the data in the study and takes responsibility for the integrity of the data and the accuracy of the data analysis.

### PEER REVIEW

The peer review history for this article is available at https://publons.com/publon/10.1111/dom.14610.

## Supporting information


**Table S1** Identifying adverse events of interest.
**Table S2**: Definitions of candidate variables included for selection.
**Table S3**: Characteristics of 11 683 participants included in the analysis, before and after imputation of missing data.
**Table S4**: Number (%) of participants included in analysis with missing EQ‐5D response.
**Table S5**: EQ‐5D‐5L questionnaire response from 11 683 participants, before and after imputation.
**Table S6**: Mean (SD) of EQ‐5D utility by number of comorbid adverse events.
**Table S7**: Mean (SD) of EQ‐5D utility by adverse event and time since event occurrence.
**Table S8**: EQ‐5D utility of patients with diabetes associated with patient characteristics, clinical risk factors and adverse events.
**Table S9**: EQ‐5D utility of patients with diabetes associated with patient characteristics, clinical risk factors and adverse events, with (a) all adverse events included and (b) using DSU tariffs.
**Table S10**: Comparison of quality of life decrements associated with diabetes‐related complications across diabetes studies.
**Table S11**: Comparison of quality of life decrements associated with bleeding events across studies.
**Figure S1**: Marginal effect of age on EQ‐5D utility.
**Appendix S1** Further details on statistical methods.

## Data Availability

Data sharing is not applicable to this article as no new data were created or analyzed in this study.
