# Supplementary material for: Decrements in health‐related quality of life associated with adverse events in people with diabetes
Source: Diabetes Obes Metab. 2021 Dec 20;24(3):530–8. doi: 10.1111/dom.14610 (PMC9361007; doi:10.1111/dom.14610)
Supplement: Supplementary file 1 — Table S1 Identifying adverse events of interest. Table S2: Definitions of candidate variables included for selection. Table S3: Characteristics of 11 683 participants included in the analysis, before and after imputation of missing data. Table S4: Number (%) of participants included in analysis with missing EQ‐5D response. Table S5: EQ‐5D‐5L questionnaire response from 11 683 participants, before and after imputation. Table S6: Mean (SD) of EQ‐5D utility by number of comorbid adverse events. Table S7: Mean (SD) of EQ‐5D utility by adverse event and time since event occurrence. Table S8: EQ‐5D utility of patients with diabetes associated with patient characteristics, clinical risk factors and adverse events. Table S9: EQ‐5D utility of patients with diabetes associated with patient characteristics, clinical risk factors and adverse events, with (a) all adverse events included and (b) using DSU tariffs. Table S10: Comparison of quality of life decrements associated with diabetes‐related complications across diabetes studies. Table S11: Comparison of quality of life decrements associated with bleeding events across studies. Figure S1: Marginal effect of age on EQ‐5D utility. Appendix S1 Further details on statistical methods. [file DOM-24-530-s001.docx]

**Decrements in health-related quality of life associated with adverse events in people with diabetes**

Supplementary Material

Contents

[Supplementary Tables 2](#_Toc87438450)

[Supplementary Table S1: Identifying adverse events of interest 2](#_Toc87438451)

[Supplementary Table S2: Definitions of candidate variables included for selection 4](#_Toc87438452)

[Supplementary Table S3: Characteristics of 11683 participants included in the analysis, before and after imputation of missing data 6](#_Toc87438453)

[Supplementary Table S4: Number (%) of participants included in analysis with missing EQ-5D response 9](#_Toc87438454)

[Supplementary Table S5: EQ-5D-5L questionnaire response from 11683 participants, before and after imputation 10](#_Toc87438455)

[Supplementary Table S6: Mean (SD) of EQ-5D utility by number of comorbid adverse events 12](#_Toc87438456)

[Supplementary Table S7: Mean (SD) of EQ-5D utility by adverse event and time since event occurrence 13](#_Toc87438457)

[Supplementary Table S8: EQ-5D utility of patients with diabetes associated with patient characteristics, clinical risk factors and adverse events 15](#_Toc87438458)

[Supplementary Table S9: EQ-5D utility of patients with diabetes associated with patient characteristics, clinical risk factors and adverse events, with (a) all adverse events included and (b) using DSU tariffs 17](#_Toc87438459)

[Supplementary Table S10: Comparison of quality of life decrements associated with diabetes-related complications across diabetes studies 19](#_Toc87438460)

[Supplementary Table S11: Comparison of quality of life decrements associated with bleeding events across studies 22](#_Toc87438461)

[Supplementary Figures 23](#_Toc87438462)

[Supplementary Figure S1: Marginal effect of age on EQ-5D utility 23](#_Toc87438463)

[Supplementary Section 1: Further details on statistical methods 24](#_Toc87438464)

## Supplementary Tables

### Supplementary Table S1: Identifying adverse events of interest

|  | Adverse events in trial | Hospital Episode Statistics data |
| --- | --- | --- |
| **Cardiovascular disease** |  |  |
| Myocardial infarction | ✓^†^  Evidence of cardiac necrosis and other evidence of acute MI and no other diagnosis was likely. Silent MI not included. |  |
| Coronary revascularisation (urgent) | ✓^†^  Coronary angioplasty, stenting or coronary artery bypass grafting. |  |
| Coronary revascularisation (non-urgent) | ✓^†^  Coronary angioplasty, stenting or coronary artery bypass grafting. |  |
| Transient ischaemic attack | ✓^†^  Transient episode of neurological dysfunction, lasting less than 24 hours, without clear evidence of acute infarction, haemorrhage, trauma or other cause. |  |
| Ischaemic stroke | ✓^†^  Acute symptomatic episode of focal or global neurological dysfunction as a result of infarction which lasted >24 hours, lead to death, or was associated with imaging evidence of acute infarct corresponding with clinical syndrome |  |
| Heart failure | ✓^‡^ | ✓ ICD-10 codes in primary diagnosis position:  I50 Heart failure; I110, I130, I132 Hypertensive heart disease with (congestive) heart failure; I255, I420, I425, I426, I427, I428, I429, I430, I431, I432, I438 Congestive cardiomyopathy |
| **Bleeding** |  |  |
| Intracranial haemorrhage | ✓^†^  Includes spontaneous intracranial bleeds or those associated with injury in the absence of major trauma |  |
| Gastrointestinal bleed | ✓^†^ |  |
| Other major bleed | ✓^†^  Major bleeds that required hospitalisation or transfusion, or was fatal or disabling. Excludes eye bleeds |  |
| **Cancer** |  |  |
| Gastrointestinal tract cancer | ✓^†^ |  |
| Non-gastrointestinal tract cancer | ✓^†^  Excludes non-melanoma skin cancer |  |
| **Microvascular disease** |  |  |
| Amputation | ✓^‡^  Lower limb amputation | ✓ OPCS-4 codes: X09 Amputation of leg; X10 Amputation of foot; X11 Amputation of toe |
| End-stage renal disease | ✓^‡^  Includes participants who had dialysis or renal transplant | ✓ Algorithm & ICD-10 codes from Bush et al. (2017)^1^ |

^†^Events corresponding to these endpoints were sought for and adjudicated in the ASCEND study.^2^ The date of event occurrence recorded in the study was used.

^‡^These endpoints were not sought for nor adjudicated in ASCEND. Hospital episode data was used to supplement the identification of these events. Earliest recorded date between the two sources used.

References:

1. Kathryn Bush, John Nolan, Qiuli Zhang, Will Herrington, Cathie Sudlow. Definitions of End Stage Renal Disease for UK Biobank Phase 1 Outcomes Adjudication. Published August 2017. Accessed May 7, 2021. <https://biobank.ndph.ox.ac.uk/ukb/ukb/docs/alg_outcome_esrd.pdf>
2. The ASCEND Study Collaborative Group. Effects of Aspirin for Primary Prevention in Persons with Diabetes Mellitus. *N Engl J Med*. 2018;379(16):1529-1539. doi:10.1056/NEJMoa1804988

### Supplementary Table S2: Definitions of candidate variables included for selection

| Variables | Categories (if categorical) | Note |
| --- | --- | --- |
| Diabetes type | Type 1  Type 2 |  |
| Sex | Male  Female |  |
| Smoking status | Current smoker  Former/never smoker |  |
| Race | White  Indian/Pakistani/Bangladeshi  African/Caribbean |  |
| Townsend Index | Q1: <-2.42  Q2: >=-2.42, <-0.44  Q3: >=-0.44, <1.79  Q4: >= 1.79, <4.75  Q5: >= 4.75 | Measure of socioeconomic status calculated using postcode at randomization. Higher value indicates greater deprivation. Stratified according to range of scores of 2011 UK population quintiles. |
| Hypertension | Y / N |  |
| Diabetic retinopathy | Y / N |  |
| Use of statin | Y / N |  |
| Use of ACE inhibitor or ARB | Y / N |  |
| Age (years) |  | At time of EQ-5D questionnaire response. |
| Diabetes duration (years) | <10  >=10, <15  >=15, <25  >=25 | At time of EQ-5D questionnaire response. |
| BMI (kg/m^2^) | <25  >=25, <30  >=30, <35  >=35 | Based on self-reported height and weight |
| HbA1c (IFCC mmol/mol) | < 48  >= 48, < 64  >= 64 |  |
| HDL cholesterol (mmol/L) | < 1.0  >= 1.0, < 1.5  >= 1.5 |  |
| Non-HDL cholesterol (mmol/L) | < 2.5  >= 2.5, < 3.5  >= 3.5 |  |
| Systolic blood pressure  (mmHg) | <130  >=130, <140  >=140 |  |
| Diastolic blood pressure  (mmHg) | <75  >=75, <85 |  |
| Urinary albumin/creatinine ratio (mg/mmol) | < 3  >=3 |  |
| eGFR (ml/min/1.73m^2^) | < 45  >= 45, < 60  >= 60, < 90  >=90 | Calculated from blood cystatin C concentration using the CKD-EPI formula |
| Disease history  (adverse events listed below) | No history  <=1 yr  > 1yr | Time since event occurrence relative to date of questionnaire response. <=1yr, event occurred within one year prior to EQ-5D questionnaire response; >1yr, event occurred more than a year prior to EQ-5D questionnaire response. |
| Myocardial infarction |  |  |
| Coronary revascularisation  (non-urgent) |  |  |
| Coronary revascularisation  (urgent) |  |  |
| Transient ischaemic attack |  |  |
| Ischaemic stroke |  |  |
| Heart failure |  |  |
| GI tract cancer |  |  |
| Non-GI tract cancer |  |  |
| Intracranial haemorrhage |  |  |
| GI bleed |  |  |
| Other major bleed |  | Other major bleed refers to bleeding events that are not intracranial haemorrhage or GI bleed. |
| Amputation |  |  |
| End-stage renal disease |  |  |

BMI, body mass index; HbA1c, glycated haemoglobin; IFCC, International Federation of Clinical Chemistry; HDL, High-density lipoprotein; eGFR, estimated glomerular filtration rate; GI, gastrointestinal.

Age, diabetes duration and disease histories were at time of EQ-5D questionnaire response. Other patient characteristics and clinical risk factors were measured at recruitment into the ASCEND study.

### Supplementary Table S3: Characteristics of 11683 participants included in the analysis, before and after imputation of missing data

|  | Before imputation | After imputation |
| --- | --- | --- |
| ***Participant characteristics at entry into the ASCEND study*** | | |
| **Diabetes type** |  |  |
| Type 1 | 707 (6.1%) | 707 (6.1%) |
| Type 2 | 10976 (93.9%) | 10976 (93.9%) |
| **Sex** |  |  |
| Male | 7347 (62.9%) | 7347 (62.9%) |
| Female | 4336 (37.1%) | 4336 (37.1%) |
| **Smoking status** |  |  |
| Current smoker | 813 (7.0%) | 813 (7.0%) |
| Former/Never smoker | 10734 (91.9%) | 10870 (93.0%) |
| Missing | 136 (1.2%) |  |
| **Race** |  |  |
| White | 11282 (96.6%) | 11450 (98.0%) |
| Indian/Pakistani/Bangladeshi | 147 (1.3%) | 147 (1.3%) |
| African/Caribbean | 86 (0.7%) | 86 (0.7%) |
| Missing | 168 (1.4%) |  |
| **Townsend Index^†^** |  |  |
| Q1: <-2.42 (least deprived) | 5232 (44.8%) | 5258 (45.0%) |
| Q2: >=-2.42, <-0.44 | 2934 (25.1%) | 2934 (25.1%) |
| Q3: >=-0.44, < 1.79 | 1851 (15.8%) | 1851 (15.8%) |
| Q4: >= 1.79, < 4.75 | 1237 (10.6%) | 1237 (10.6%) |
| Q5: >= 4.75 (most deprived) | 403 (3.4%) | 403 (3.4%) |
| Missing | 26 (0.2%) |  |
| **Hypertension** |  |  |
| Y | 7173 (61.4%) | 7248 (62.0%) |
| N | 4435 (38.0%) | 4435 (38.0%) |
| Missing | 75 (0.6%) |  |
| **Diabetic retinopathy** |  |  |
| Y | 2232 (19.1%) | 2232 (19.1%) |
| N | 9359 (80.1%) | 9451 (80.9%) |
| Missing | 92 (0.8%) |  |
| **Use of statin** |  |  |
| Y | 8919 (76.3%) | 8919 (76.3%) |
| N | 2764 (23.7%) | 2764 (23.7%) |
| **Use of ACE Inhibitor or ARB** |  |  |
| Y | 6818 (58.4%) | 6818 (58.4%) |
| N | 4865 (41.6%) | 4865 (41.6%) |
| **Age (years)** |  |  |
| Mean (SD) | 62.8 (8.5) | 62.3 (8.5) |
| <50 | 757 (6.5%) | 757 (6.5%) |
| >=50, <60 | 3542 (30.3%) | 3542 (30.3%) |
| >=60, <70 | 5024 (43%) | 5024 (43%) |
| >=70 | 2360 (20.2%) | 2360 (20.2%) |
| **Diabetes duration (years)** |  |  |
| Mean (SD) | 9.7 (9.3) | 9.6 (9.2) |
| <5 | 3765 (32.2%) | 3867 (33.1%) |
| >=5, <10 | 3348 (28.7%) | 3680 (31.5%) |
| >=10, <20 | 2608 (22.3%) | 2730 (23.4%) |
| >=20 | 1374 (11.8%) | 1406 (12%) |
| Missing | 588 (5%) |  |
| **Body-mass index (kg/m^2^)** |  |  |
| Mean (SD) | 31.0 (6.4) | 31.0 (6.4) |
| <25 | 1539 (13.2%) | 1540 (13.2%) |
| >=25, <30 | 4224 (36.2%) | 4269 (36.5%) |
| >=30, <35 | 3426 (29.3%) | 3456 (29.6%) |
| >=35 | 2412 (20.6%) | 2418 (20.7%) |
| Missing | 82 (0.7%) |  |
| **HbA1c (IFCC mmol/mol)** |  |  |
| Mean (SD) | 54.4 (12.4) | 54.6 (10.9) |
| < 48 | 2532 (21.7%) | 3134 (26.8%) |
| >= 48, < 64 | 3625 (31.0%) | 6653 (56.9%) |
| >= 64 | 1361 (11.6%) | 1896 (16.2%) |
| Missing | 4165 (35.7%) |  |
| **HDL cholesterol (mmol/L)** |  |  |
| Mean (SD) | 1.27 (0.35) | 1.27 (0.29) |
| < 1.0 | 1630 (14.0%) | 1655 (14.2%) |
| >= 1.0, < 1.5 | 4206 (36.0%) | 8243 (70.6%) |
| >= 1.5 | 1672 (14.3%) | 1785 (15.3%) |
| Missing | 4175 (35.7%) |  |
| **Non-HDL cholesterol (mmol/L)** |  |  |
| Mean (SD) | 2.88 (0.83) | 2.88 (0.68) |
| < 2.5 | 2650 (22.7%) | 2902 (24.8%) |
| >= 2.5, < 3.5 | 3398 (29.1%) | 7247 (62.0%) |
| >= 3.5 | 1460 (12.5%) | 1534 (13.1%) |
| Missing | 4175 (35.7%) |  |
| **Systolic blood pressure (mmHg)** |  |  |
| Mean (SD) | 135.9 (15.0) | 135.8 (13.2) |
| <130 | 2622 (22.4%) | 3233 (27.7%) |
| >=130, <140 | 2390 (20.5%) | 4283 (36.7%) |
| >=140 | 3479 (29.8%) | 4167 (35.7%) |
| Missing | 3192 (27.3%) |  |
| **Diastolic blood pressure (mmHg)** |  |  |
| Mean (SD) | 77.3 (9.3) | 77.5 (8.2) |
| <75 | 3149 (27.0%) | 3834 (32.8%) |
| >=75, <85 | 3650 (31.2%) | 6004 (51.4%) |
| >=85 | 1687 (14.4%) | 1845 (15.8%) |
| Missing | 3197 (27.4%) |  |
| **Urinary albumin/creatinine ratio (mg/mmol) ^‡^** |  |  |
| < 3 | 6644 (56.9%) | 10481 (89.7%) |
| >= 3 | 854 (7.3%) | 1202 (10.3%) |
| Missing | 4185 (35.8%) |  |
| **eGFR (ml/min/1.73m^2^)^§^** |  |  |
| < 45 | 209 (1.8%) | 243 (2.1%) |
| >= 45, < 60 | 584 (5.0%) | 817 (7.0%) |
| >= 60, < 90 | 3081 (26.4%) | 4636 (39.7%) |
| >= 90 | 3645 (31.2%) | 5987 (51.2%) |
| Missing | 4164 (35.6%) |  |
| ***Participant characteristics at EQ-5D questionnaire response*** | | |
| **Age (years)** |  |  |
| Mean (SD) | 68.5 (8.3) | 68.5 (8.3) |
| <60 | 1693 (14.5%) | 1693 (14.5%) |
| >=60, <70 | 4856 (41.6%) | 4856 (41.6%) |
| >=70, <80 | 3978 (34%) | 3978 (34%) |
| >=80 | 1156 (9.9%) | 1156 (9.9%) |
| **Diabetes duration (years)** |  |  |
| Mean (SD) | 16.4 (9.4) | 16.4 (9.6) |
| <10 | 2732 (23.4%) | 2769 (23.7%) |
| >=10, <15 | 3663 (31.4%) | 3999 (34.2%) |
| >=15, <25 | 3131 (26.8%) | 3303 (28.3%) |
| >=25 | 1569 (13.4%) | 1612 (13.8%) |

Summary values are N (%) unless otherwise specified. HbA1c, glycated haemoglobin; IFCC, International Federation of Clinical Chemistry; HDL, High-density lipoprotein; eGFR, estimated glomerular filtration rate; ACE, Angiotensin converting enzyme; ARB, Angiotensin II receptor blocker.

Only 7589 (65%) participants returned usable blood/urine sample in the study.

^†^Townsend index stratified according to range of scores of 2011 UK population quintiles.

^‡^1896 (25%) participants who had undetectable albumin levels were reclassified as having no albuminuria (urinary albumin/creatinine ratio <3).

^§^Calculated from blood cystatin C concentration using the CKD-EPI formula.

### Supplementary Table S4: Number (%) of participants included in analysis with missing EQ-5D response

| EQ-5D response | N (%) |
| --- | --- |
| Did not respond | 436 (3.7%) |
| Returned response | 11247 (96.3%) |
| No. of domains missing | |
| 0 | 11028 (98.1%) |
| 1 | 144 (1.3%) |
| 2 | 30 (0.3%) |
| 3 | 15 (0.1%) |
| 4 | 9 (0.1%) |
| 5 | ^†^20 (0.2%) |

^†^Of the 20 participants who responded to the questionnaire with all 5 domains missing, 9 had VAS score.

### Supplementary Table S5: EQ-5D-5L questionnaire response from 11683 participants, before and after imputation

|  | Before imputation | After imputation |
| --- | --- | --- |
| **EQ-5D utility** |  |  |
| Mean (SD) | 0.773 (0.220) | 0.771 (0.221) |
| Missing | 655 (5.6%) |  |
| **EQ-5D VAS score** |  |  |
| Mean (SD) | 77.3 (17.9) | 77.3 (17.9) |
| Missing | 498 (4.3%) |  |
| **Reported full health^†^** |  |  |
| Yes | 3110 (26.6%) | 3258 (27.9%) |
| No | 7918 (67.8%) | 8425 (72.1%) |
| Missing | 655 (5.6%) |  |
| ***EQ-5D dimensions*** |  |  |
| **Mobility** |  |  |
| 1 | 6212 (53.2%) | 6466 (55.3%) |
| 2 | 2453 (21.0%) | 2584 (22.1%) |
| 3 | 1641 (14.0%) | 1720 (14.7%) |
| 4 | 836 (7.2%) | 871 (7.5%) |
| 5 | 39 (0.3%) | 42 (0.4%) |
| Missing | 502 (4.3%) |  |
| **Self-care** |  |  |
| 1 | 9494 (81.3%) | 9925 (85%) |
| 2 | 970 (8.3%) | 1023 (8.8%) |
| 3 | 545 (4.7%) | 571 (4.9%) |
| 4 | 137 (1.2%) | 147 (1.3%) |
| 5 | 16 (0.1%) | 17 (0.1%) |
| Missing | 521 (4.5%) |  |
| **Usual activities** |  |  |
| 1 | 6868 (58.8%) | 7156 (61.3%) |
| 2 | 2264 (19.4%) | 2380 (20.4%) |
| 3 | 1430 (12.2%) | 1503 (12.9%) |
| 4 | 492 (4.2%) | 516 (4.4%) |
| 5 | 119 (1.0%) | 128 (1.1%) |
| Missing | 510 (4.4%) |  |
| **Pain/Discomfort** |  |  |
| 1 | 4204 (36%) | 4392 (37.6%) |
| 2 | 4041 (34.6%) | 4214 (36.1%) |
| 3 | 2053 (17.6%) | 2155 (18.4%) |
| 4 | 768 (6.6%) | 805 (6.9%) |
| 5 | 111 (1.0%) | 117 (1%) |
| Missing | 506 (4.3%) |  |
| **Anxiety/Depression** |  |  |
| 1 | 7905 (67.7%) | 8255 (70.7%) |
| 2 | 2133 (18.3%) | 2249 (19.3%) |
| 3 | 923 (7.9%) | 981 (8.4%) |
| 4 | 144 (1.2%) | 148 (1.3%) |
| 5 | 47 (0.4%) | 50 (0.4%) |
| Missing | 531 (4.5%) |  |

Summary values are N (%) unless otherwise specified. The five levels of EQ-5D-5L are defined as follows: 1 no problem, 2 slight problems, 3 moderate problems, 4 severe problems, 5 extreme problems/unable to.

^†^ Full health refers to rating 1 for all EQ-5D dimensions.

### Supplementary Table S6: Mean (SD) of EQ-5D utility by number of comorbid adverse events

| No. of comorbid events | N (%) | Mean (SD) of EQ-5D utility | |
| --- | --- | --- | --- |
|  |  | van Hout tariff | DSU tariff |
| 0 | 9970 (85.3%) | 0.781 (0.216) | 0.776 (0.216) |
| 1 | 1375 (11.8%) | 0.722 (0.241) | 0.715 (0.243) |
| 2 | 267 (2.3%) | 0.683 (0.261) | 0.677 (0.261) |
| >= 3 | 71 (0.6%) | 0.648 (0.236) | 0.638 (0.252) |

The most frequent co-occurring events among ASCEND participants were myocardial infarction and urgent coronary revascularisation (N = 140), followed by stroke and TIA (N = 44). There were fewer than 20 occasions for other co-occurring events.

### Supplementary Table S7: Mean (SD) of EQ-5D utility by adverse event and time since event occurrence

| Time since event occurrence | No. of participants (%) | Mean (SD) of EQ-5D utility | |  |
| --- | --- | --- | --- | --- |
|  |  | van Hout tariff | DSU tariff |  |
| Myocardial infarction | | | | |
| No history | 11494 (98.4%) | 0.77 (0.22) | 0.77 (0.22) |  |
| <=1yr | 34 (0.3%) | 0.72 (0.26) | 0.72 (0.26) |  |
| >1yr | 155 (1.3%) | 0.72 (0.24) | 0.71 (0.25) |  |
| Coronary revascularisation (non-urgent) | | | | |
| No history | 11496 (98.4%) | 0.77 (0.22) | 0.77 (0.22) |  |
| <=1yr | 28 (0.2%) | 0.78 (0.21) | 0.78 (0.19) |  |
| >1yr | 159 (1.4%) | 0.74 (0.22) | 0.73 (0.23) |  |
| Coronary revascularisation (urgent) | | | | |
| No history | 11533 (98.7%) | 0.77 (0.22) | 0.77 (0.22) |  |
| <=1yr | 25 (0.2%) | 0.70 (0.26) | 0.71 (0.25) |  |
| >1yr | 125 (1.1%) | 0.73 (0.25) | 0.72 (0.25) |  |
| Transient ischaemic attack | | | | |
| No history | 11469 (98.2%) | 0.77 (0.22) | 0.77 (0.22) |  |
| <=1yr | 38 (0.3%) | 0.64 (0.28) | 0.62 (0.30) |  |
| >1yr | 176 (1.5%) | 0.70 (0.24) | 0.68 (0.25) |  |
| Ischaemic stroke | | | | |
| No history | 11485 (98.3%) | 0.77 (0.22) | 0.77 (0.22) |  |
| <=1yr | 42 (0.4%) | 0.72 (0.20) | 0.71 (0.21) |  |
| >1yr | 156 (1.3%) | 0.68 (0.25) | 0.67 (0.25) |  |
| Heart failure | | | | |
| No history | 11578 (99.1%) | 0.77 (0.22) | 0.77 (0.22) |  |
| <=1yr | 30 (0.3%) | 0.51 (0.27) | 0.51 (0.27) |  |
| >1yr | 75 (0.6%) | 0.66 (0.24) | 0.64 (0.25) |  |
| GI tract cancer |  |  |  |  |
| No history | 11537 (98.8%) | 0.77 (0.22) | 0.77 (0.22) |  |
| <=1yr | 31 (0.3%) | 0.73 (0.23) | 0.71 (0.24) |  |
| >1yr | 115 (1.0%) | 0.73 (0.23) | 0.72 (0.24) |  |
| Non-GI tract cancer |  |  |  |  |
| No history | 11022 (94.3%) | 0.77 (0.22) | 0.77 (0.22) |  |
| <=1yr | 113 (1.0%) | 0.77 (0.22) | 0.76 (0.23) |  |
| >1yr | 548 (4.7%) | 0.74 (0.23) | 0.73 (0.23) |  |
| Intracranial haemorrhage |  |  |  |  |
| No history | 11661 (99.8%) | 0.77 (0.22) | 0.77 (0.22) |  |
| <=1yr | 2 (0.0%) | 0.49 (0.35) | 0.48 (0.41) |  |
| >1yr | 20 (0.2%) | 0.56 (0.39) | 0.60 (0.33) |  |
| GI bleed |  |  |  |  |
| No history | 11580 (99.1%) | 0.77 (0.22) | 0.77 (0.22) |  |
| <=1yr | 16 (0.1%) | 0.58 (0.27) | 0.58 (0.27) |  |
| >1yr | 87 (0.7%) | 0.64 (0.29) | 0.64 (0.29) |  |
| Other major bleed |  |  |  |  |
| No history | 11613 (99.4%) | 0.77 (0.22) | 0.77 (0.22) |  |
| <=1yr | 7 (0.1%) | 0.74 (0.23) | 0.73 (0.25) |  |
| >1yr | 63 (0.5%) | 0.65 (0.28) | 0.65 (0.29) |  |
| Amputation |  |  |  |  |
| No history | 11623 (99.5%) | 0.77 (0.22) | 0.77 (0.22) |  |
| <=1yr | 10 (0.1%) | 0.42 (0.36) | 0.41 (0.34) |  |
| >1yr | 50 (0.4%) | 0.56 (0.25) | 0.56 (0.25) |  |
| End-stage renal disease |  |  |  |  |
| No history | 11641 (99.6%) | 0.77 (0.22) | 0.77 (0.22) |  |
| <=1yr | 8 (0.1%) | 0.68 (0.14) | 0.68 (0.15) |  |
| >1yr | 34 (0.3%) | 0.64 (0.28) | 0.62 (0.29) |  |

<=1yr, event occurred within one year prior to EQ-5D questionnaire response; >1yr, event occurred more than a year prior to EQ-5D questionnaire response; GI, gastrointestinal. Other major bleed refers to bleeding events that are not intracranial haemorrhage or GI bleed.

### Supplementary Table S8: EQ-5D utility of patients with diabetes associated with patient characteristics, clinical risk factors and adverse events

| Variable | | EQ-5D utility (95% CI) |
| --- | --- | --- |
| (Intercept) | | 0.906 (0.891, 0.920)** |
| Sex (ref: Male) | | |
|  | Female | -0.023 (-0.031, -0.015)** |
| Current smoker (ref: N) | | |
|  | Y | -0.052 (-0.067, -0.037)** |
| Townsend index (ref: Q1 (least deprived)) | | |
|  | Q2 | -0.009 (-0.019, 0) |
|  | Q3 | -0.042 (-0.053, -0.03)** |
|  | Q4 | -0.069 (-0.082, -0.056)** |
|  | Q5 (most deprived) | -0.074 (-0.095, -0.053)** |
| Age at time of questionnaire (per 10 years, centered at 70) | | |
|  | Age | -0.014 (-0.02, -0.009)** |
|  | Age^2^ | -0.006 (-0.01, -0.002)** |
| BMI (ref: <25 kg/m^2^) | | |
|  | >=25, <30 | -0.033 (-0.045, -0.02)** |
|  | >=30, <35 | -0.066 (-0.079, -0.053)** |
|  | >=35 | -0.134 (-0.148, -0.12)** |
| Diabetes duration at time of questionnaire (ref: <10 years) | | |
|  | >=10, <15 | -0.011 (-0.021, 0)* |
|  | >=15, <25 | -0.026 (-0.037, -0.015)** |
|  | >=25 | -0.029 (-0.043, -0.016)** |
| eGFR (ref: >=90 ml/min/1.73m^2^) | | |
|  | >=60, <90 | -0.03 (-0.039, -0.02)** |
|  | >=45, <60 | -0.072 (-0.089, -0.054)** |
|  | <45 | -0.08 (-0.108, -0.051)** |
| Albuminuria (urinary albumin/creatinine ratio >=3 mg/mmol) (ref: N) | | |
|  | Y | -0.018 (-0.031, -0.005)** |
| Disease history (ref: no event) | | |
| Transient ischaemic attack | | |
|  | Any history | -0.057 (-0.086, -0.028)** |
| Ischaemic stroke | | |
|  | Any history | -0.062 (-0.092, -0.032)** |
| Heart failure | | |
|  | <=1yr | -0.183 (-0.258, -0.108)** |
|  | >1yr | -0.046 (-0.094, 0.001) |
| Non-GI tract cancer | | |
|  | Any history | -0.026 (-0.043, -0.01)** |
| Intracranial haemorrhage | | |
|  | Any history | -0.164 (-0.251, -0.076)** |
| GI bleed | | |
|  | Any history | -0.091 (-0.132, -0.051)** |
| Other major bleed | | |
|  | Any history | -0.096 (-0.146, -0.047)** |
| Amputation | | |
|  | Any history | -0.206 (-0.259, -0.152)** |

**: p-value < 0.01; *: p-value < 0.05. <=1yr, event occurred within one year prior to EQ-5D questionnaire response; >1yr, event occurred more than a year prior to EQ-5D questionnaire response; BMI, body mass index; eGFR, estimated glomerular filtration rate; GI, gastrointestinal. Other major bleed refers to bleeding events that are not intracranial haemorrhage or GI bleed. Number of participants experiencing each adverse event presented in brackets next to time adverse event occurrence. Intercept value gives the EQ-5D utility for the reference individual (male, not current smoker, living in least deprived region, aged 70, BMI <25kg/m^2^, diabetes duration <10 years, eGFR>=90ml/min/1.73m^2^, no albuminuria, with no disease history).

### Supplementary Table S9: EQ-5D utility of patients with diabetes associated with patient characteristics, clinical risk factors and adverse events, with (a) all adverse events included and (b) using DSU tariffs

| Variable | | EQ-5D utility (95% CI) | |
| --- | --- | --- | --- |
|  |  | (a) All adverse events included | (b) DSU EQ-5D tariff |
| (Intercept) | | 0.906 (0.892, 0.92)** | 0.899 (0.884, 0.913)** |
| Sex (ref: Male) | |  |  |
|  | Female | -0.023 (-0.032, -0.015)** | -0.018 (-0.026, -0.01)** |
| Current smoker (ref: N) | |  |  |
|  | Y | -0.052 (-0.067, -0.036)** | -0.055 (-0.07, -0.04)** |
| Townsend index (ref: Q1 (least deprived)) | | | |
|  | Q2 | -0.009 (-0.019, 0) | -0.009 (-0.019, 0) |
|  | Q3 | -0.042 (-0.053, -0.031)** | -0.043 (-0.054, -0.032)** |
|  | Q4 | -0.069 (-0.082, -0.056)** | -0.073 (-0.086, -0.06)** |
|  | Q5 (most deprived) | -0.074 (-0.096, -0.053)** | -0.076 (-0.098, -0.055)** |
| Age at time of questionnaire (per 10 years, centered at 70) | | | |
|  | Age | -0.014 (-0.02, -0.009)** | -0.016 (-0.021, -0.01)** |
|  | Age^2^ | -0.006 (-0.01, -0.002)** | -0.006 (-0.01, -0.001)* |
| BMI (ref: <25 kg/m^2^) | | | |
|  | >=25, <30 | -0.033 (-0.045, -0.02)** | -0.031 (-0.044, -0.019)** |
|  | >=30, <35 | -0.066 (-0.079, -0.053)** | -0.065 (-0.078, -0.052)** |
|  | >=35 | -0.134 (-0.148, -0.12)** | -0.131 (-0.145, -0.117)** |
| Diabetes duration at time of questionnaire (ref: <10 years) | | | |
|  | >=10, <15 | -0.01 (-0.021, 0)* | -0.011 (-0.022, -0.001)* |
|  | >=15, <25 | -0.026 (-0.036, -0.015)** | -0.026 (-0.037, -0.015)** |
|  | >=25 | -0.029 (-0.042, -0.016)** | -0.031 (-0.044, -0.018)** |
| eGFR (ref: >=90 ml/min/1.73m^2^) | | | |
|  | >=60, <90 | -0.029 (-0.039, -0.02)** | -0.03 (-0.04, -0.021)** |
|  | >=45, <60 | -0.071 (-0.089, -0.054)** | -0.073 (-0.09, -0.055)** |
|  | <45 | -0.077 (-0.106, -0.048)** | -0.083 (-0.112, -0.054)** |
| Albuminuria (urinary albumin/creatinine ratio >=3 mg/mmol) (ref: N) | | | |
|  | Y | -0.017 (-0.03, -0.004)* | -0.018 (-0.031, -0.005)** |
| Disease history (ref: no event) | | | |
| Myocardial infarction | |  |  |
|  | Any history | -0.019 (-0.073, 0.035) |  |
| Coronary revascularisation (non-urgent) | | | |
|  | Any history | -0.007 (-0.038, 0.023) |  |
| Coronary revascularisation (urgent) | | | |
|  | Any history | -0.004 (-0.064, 0.057) |  |
| Transient ischaemic attack | |  |  |
|  | Any history | -0.057 (-0.086, -0.028)** | -0.066 (-0.095, -0.037)** |
| Ischaemic stroke | |  |  |
|  | Any history | -0.061 (-0.091, -0.031)** | -0.061 (-0.091, -0.03)** |
| Heart failure | |  |  |
|  | <=1yr | -0.179 (-0.254, -0.104)** | -0.184 (-0.26, -0.109)** |
|  | >1yr | -0.043 (-0.091, 0.005) | -0.052 (-0.1, -0.004)* |
| GI tract cancer | |  |  |
|  | Any history | -0.015 (-0.053, 0.022) |  |
| Non-GI tract cancer | |  |  |
|  | Any history | -0.026 (-0.042, -0.009)** | -0.028 (-0.045, -0.012)** |
| Intracranial haemorrhage | |  |  |
|  | Any history | -0.162 (-0.25, -0.074)** | -0.133 (-0.221, -0.045)** |
| GI bleed | |  |  |
|  | Any history | -0.089 (-0.129, -0.048)** | -0.087 (-0.128, -0.046)** |
| Other major bleed | |  |  |
|  | Any history | -0.095 (-0.144, -0.045)** | -0.096 (-0.146, -0.047)** |
| Amputation | |  |  |
|  | Any history | -0.204 (-0.257, -0.15)** | -0.195 (-0.249, -0.141)** |
| End-stage renal disease | |  |  |
|  | Any history | -0.036 (-0.102, 0.029) |  |

**: p-value < 0.01; *: p-value < 0.05. <=1yr, event occurred within one year prior to EQ-5D questionnaire response; >1yr, event occurred more than a year prior to EQ-5D questionnaire response; BMI, body mass index; eGFR, estimated glomerular filtration rate; GI, gastrointestinal. Other major bleed refers to bleeding events that are not intracranial haemorrhage or GI bleed. Number of participants experiencing each adverse event presented in brackets next to time adverse event occurrence. Intercept values give the EQ-5D utility for the reference individual (male, not current smoker, living in least deprived region, aged 70, BMI <25kg/m^2^, diabetes duration <10 years, eGFR>=90ml/min/1.73m^2^, no albuminuria, with no disease history).

### Supplementary Table S10: Comparison of quality of life decrements associated with diabetes-related complications across diabetes studies

|  |  | ASCEND | ACCORD and Look AHEAD (Neuwahl et al., 2021) | ACCORD (Shao et al., 2019) | | LEADER (Nauck et al., 2018) | SAVOR-TIMI 53 (Briggs et al., 2017) | ADVANCE (Hayes et al., 2016) | MEPS (Sullivan & Ghushchyan, 2016) | UKPDS (Alva et al., 2014) | TRIAD (Zhang et al., 2012) | Review (Beaudet et al., 2014) |
| --- | --- | --- | --- | --- | --- | --- | --- | --- | --- | --- | --- | --- |
| No. of participants |  | 11,683 | 15,252 | 8,713 | | 3,014 | 16,488 | 11,130 | 20,705 | 3,380 | 7,327 |  |
| Repeated measures of QoL |  | N | Y | Y | | Y | Y | Y | N | Y | N |  |
| Years QoL survey conducted |  | 2016/2017 | 2001 – 2012 | 2001 – 2012 | | 2010 - 2015 | 2010 - 2013 | 2001 - 2008 | 2000-2011 | 1996 - 2007 | 2000/2001 |  |
| Previous CVD |  | 0% | 35% (ACCORD)  14% (Look AHEAD) | 35% | | 81% | 79% | 32% |  |  | 31% |  |
| Instrument |  | EQ-5D | HUI-3 | HUI-3 | | EQ-5D | EQ-5D | EQ-5D | EQ-5D;  SF-12 | EQ-5D | EQ-5D |  |
| Model |  | OLS | FE | OLS | FE | Mixed effects | Descriptive | FE | Median regression | FE | OLS |  |
| Disease history (ref: no event) |  |  |  |  |  |  |  |  |  |  |  |  |
| Myocardial infarction | Any history | (NS) |  |  |  | 0.004 (NS) | -0.051 | -0.026 | -0.047 |  | -0.019 (CHD other than HF) | -0.055 |
|  | <=1yr |  | -0.028 | -0.042 | -0.018 |  |  |  |  | -0.065 |  |  |
|  | >1yr |  | -0.006 (NS) | -0.011 (NS) |  |  |  |  |  | 0.008 (NS) |  |  |
| Revascularisation | Any history | (NS) |  |  |  |  |  |  |  |  |  |  |
|  | <=1yr |  | -0.005 (NS) | -0.038 | -0.013 |  |  |  |  |  |  |  |
|  | >1yr |  | -0.001 (NS) | -0.016 |  |  |  |  |  |  |  |  |
| TIA | Any history | -0.057  (-0.086, -0.028) |  |  |  |  |  |  | -0.070 |  | -0.029 |  |
|  | <=1yr |  |  |  |  |  |  |  |  |  |  |  |
|  | >1yr |  |  |  |  |  |  |  |  |  |  |  |
| Stroke | Any history | IS: -0.062  (-0.092, -0.032)  HS: -0.164  (-0.251, -0.076)  Combined: ~ -0.082^†^ |  |  |  | -0.046 | -0.111 | -0.099 | -0.060 | -0.165 | -0.035 | -0.164 |
|  | <=1yr |  | -0.109 | -0.204 | -0.202 |  |  |  |  |  |  |  |
|  | >1yr |  | -0.051 | -0.101 |  |  |  |  |  |  |  |  |
| Heart failure^*^ | Any history |  |  |  |  | -0.055 | -0.065 | -0.045 | -0.050 | -0.101 | -0.042 | -0.108 |
|  | <=1yr | -0.183  (-0.258, -0.108) | -0.051 | -0.089 | -0.067 |  |  |  |  |  |  |  |
|  | >1yr | -0.046  (-0.094, 0.001) (NS) | -0.041 | -0.041 |  |  |  |  |  |  |  |  |
| Amputation | Any history | -0.206  (-0.259, -0.152) |  |  |  |  |  | -0.122 | -0.095 | -0.172 | -0.108 | -0.280 |
|  | <=1yr |  | -0.092 |  |  |  |  |  |  |  |  |  |
|  | >1yr |  | -0.150 |  |  |  |  |  |  |  |  |  |
| ESRD | Any history | (NS) |  | -0.024 | -0.029 |  |  | -0.049 | -0.038 (nephropathy including ESRD) |  | -0.06 | -0.164 |
|  | <=1yr |  | -0.039 |  |  |  |  |  |  |  |  |  |
|  | >1yr |  | -0.015 |  |  |  |  |  |  |  |  |  |

NS, Effect is not statistically significant at p < 0.05; TIA, transient ischaemic attack; IS, Ischaemic stroke; HS, haemorrhagic stroke / intracerebral haemorrhage; HF, heart failure; ESRD, end-stage renal disease; CHD, coronary heart disease. <=1yr, event occurred within one year prior to EQ-5D questionnaire response; >1yr, event occurred more than a year prior to EQ-5D questionnaire response.

*Refers to hospitalisation for heart failure in ACCORD, Look AHEAD, LEADER, ADVANCE, SAVOR-TIMI 53 studies.

^†^The combined estimate of QoL decrement associated with stroke (IS + HS) was calculated assuming 80% of strokes are ischaemic strokes.

### Supplementary Table S11: Comparison of quality of life decrements associated with bleeding events across studies

|  |  | ASCEND | TRANSLATE-ACS (Amin et al., 2016) | ENGAGE AF‐TIMI 48 (Wang et al., 2017) |
| --- | --- | --- | --- | --- |
| No. of participants |  | 11683 | 9290 | 10706 |
| Study population |  | Patients with diabetes without previous vascular disease, treatment arm assigned to aspirin use | Patients on dual antiplatelet therapies after percutaneous coronary intervention for acute myocardial infarction | Patients with atrial fibrillation on antiplatelet therapies |
| Repeated measures |  | N | Y | Y |
| Years QoL survey conducted |  | 2016/2017 | 2010 – 2012 | 2008 – 2013 |
| Instrument |  | EQ-5D | EQ-5D | EQ-5D |
| Model |  | OLS | Mixed effect | Mixed effect |
| Disease history (ref: no event) |  |  |  |  |
| Any major bleed | Any history |  |  |  |
|  | <=1yr |  | BARC 2-4; at 6 months: -0.0381  BARC 3-4; at 6 months: -0.0445 |  |
|  | >1yr |  |  |  |
| Intracranial haemorrhage | Any history | -0.164 (-0.251, -0.076) |  |  |
|  | <=1yr |  |  |  |
|  | >1yr |  |  |  |
| Gastrointestinal bleed | Any history | -0.091 (-0.132, -0.051) |  |  |
|  | <=1yr |  |  | At 3 months: -0.029 |
|  | >1yr |  |  | At 12 months: -0.010 |
| Other major bleed | Any history | -0.096 (-0.146, -0.047) |  |  |
|  | <=1yr |  |  | At 3 months: -0.029 |
|  | >1yr |  |  | At 12 months: (NS) |

BARC, Bleeding Academic Research Consortium’s definition for bleeding types (type 2 and above are “actionable” bleeds requiring healthcare intervention). <=1yr, event occurred within one year prior to EQ-5D questionnaire response; >1yr, event occurred more than a year prior to EQ-5D questionnaire response. Other major bleed refers to bleeding events that are not intracranial haemorrhage or GI bleed. In ASCEND, other major bleed also excludes eye bleeds.

## Supplementary Figures

### Supplementary Figure S1: Marginal effect of age on EQ-5D utility


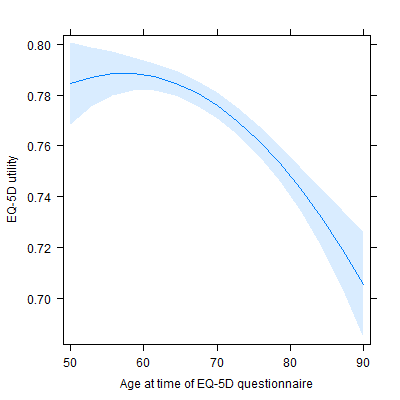


## Supplementary Section 1: Further details on statistical methods

**Non-linearity of age**

In the model including all covariates, the martingale residual plot of age suggested that there might be a non-linear relationship between age and health utility (Supplementary Figure S2). A natural cubic spline was fitted to explore the shape of the relationship. The shape of the spline fitted suggested that the polynomial form with degree 2 or a linear spline with a knot at 70 might be appropriate functional forms for age (Supplementary Figure S3), both of which are easy to compute. The marginal effect of the polynomial form of age seems to match that of the natural cubic spline most closely (Supplementary Table S11). In terms of performance metric (Supplementary Table S12), all three models performed similarly. Hence the polynomial form was selected.

| Supplementary Figure S2: Martingale residual plot | Supplementary Figure S3: Marginal effect plot (natural cubic spline) |
| --- | --- |
| 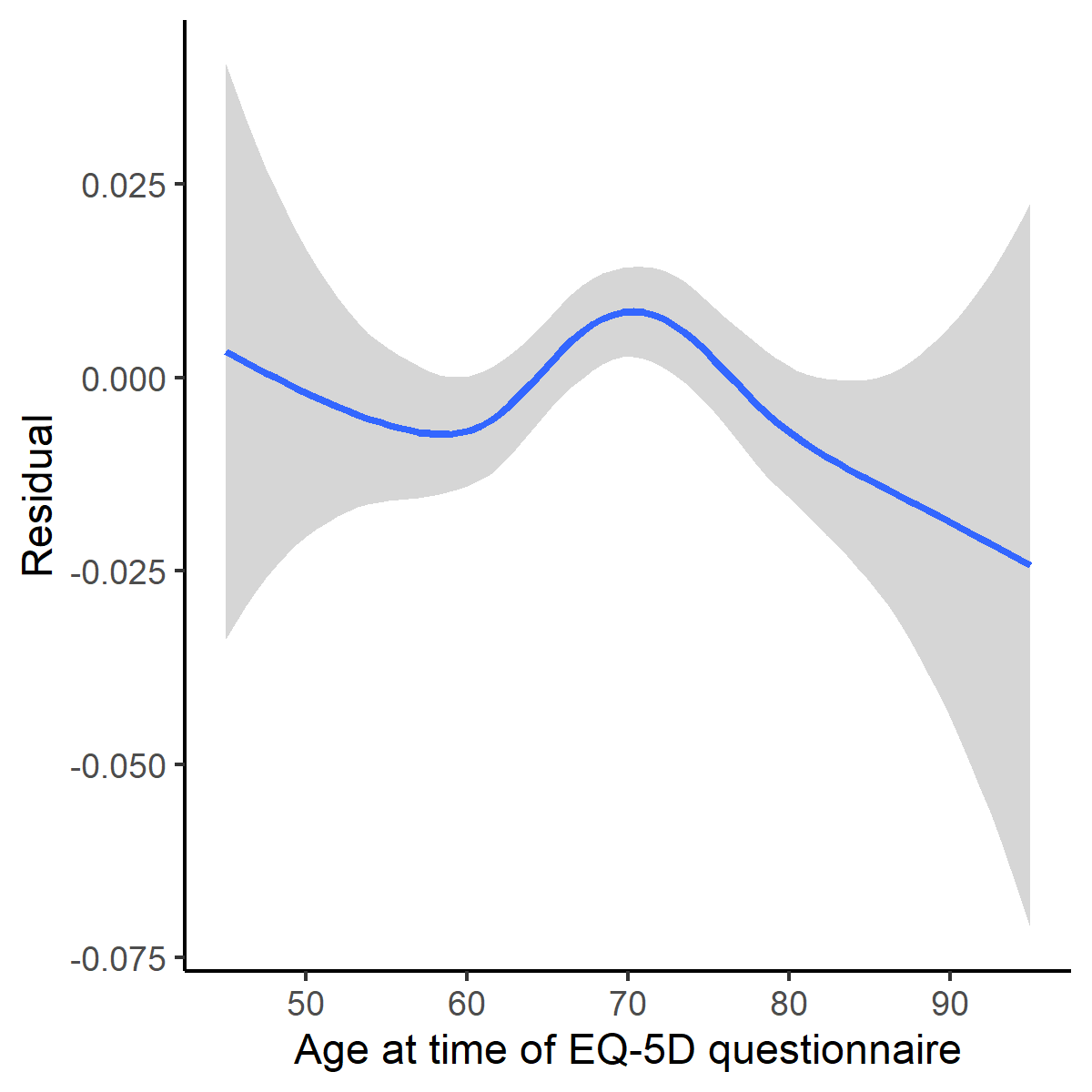 | 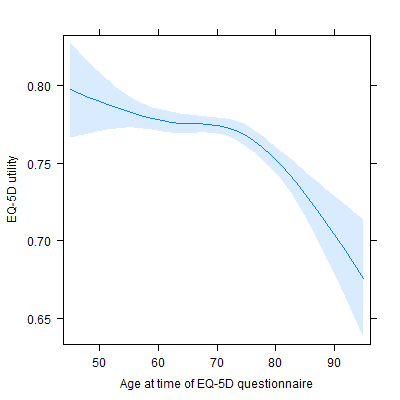 |

#### Supplementary Table S12: Marginal effects of age on EQ-5D utility

|  | Marginal effect of age relative to 70 years old | | | | | | |
| --- | --- | --- | --- | --- | --- | --- | --- |
| Form of age | 55 | 60 | 65 | 70 | 75 | 80 | 85 |
| Linear | 0.020 | 0.013 | 0.007 | 0.000 | -0.007 | -0.013 | -0.020 |
| Natural cubic spline | 0.008 | 0.003 | 0.001 | 0.000 | -0.007 | -0.022 | -0.044 |
| Polynomial degree 2 | 0.010 | 0.009 | 0.006 | 0.000 | -0.009 | -0.021 | -0.036 |
| Linear spline, knot at 70 | 0.001 | 0.000 | 0.000 | 0.000 | -0.016 | -0.031 | -0.047 |

#### Supplementary Table S13: Predictive performance of models using different forms of age

| Form of age | MSE | RMSE | MAPE | MPE | R^2^ |
| --- | --- | --- | --- | --- | --- |
| Linear | 0.04353 | 0.20864 | 0.15311 | 2.04 x 10^-14^ | 0.11083 |
| Natural cubic spline | 0.04349 | 0.20853 | 0.15310 | 1.84 x 10^-14^ | 0.11175 |
| Polynomial degree 2 | 0.04350 | 0.20858 | 0.15309 | 2.10 x 10^-14^ | 0.11137 |
| Linear spline, knot at 70 | 0.04348 | 0.20851 | 0.15305 | 2.09 x 10^-14^ | 0.11194 |

MSE: mean squared error; RMSE: root mean squared error; MAPE: mean absolute prediction error; MPE: mean prediction error; R^2^: Efron’s R^2^, square of the correlation between the predicted and observed values

**Model selection**

Given the features of EQ-5D utility data, the candidate models considered were ordinary least squares regression (OLS), Tobit model, beta regression model, generalised linear models (GLMs), and two-part models (logistic equation for first part, GLMs for second part). The predictive performance of each model is presented in Supplementary Table S13. With the exception of the Tobit and beta regression models, all other models performed similarly. The Tobit and beta regressions models had significantly greater negative bias reflected in the mean prediction error (MPE). In particular with the Tobit model, there is increasing bias at increasing utility values (Supplementary Figure S4). The Copas test, performed with 3-fold cross validation repeated 1000 times, was used to assess out-of-sample performance of the model.^1^ Results from the Copas test (Supplementary Table S14) suggest that the two-part model with identity-gamma specification might perform slightly better than the OLS. The marginal EQ-5D decrement associated with adverse events estimated by the OLS and the two-part model with identity-gamma specification is similar (Supplementary Table S15). The OLS was chosen for parsimony. One downside of using the OLS for modelling EQ-5D utility is the possibility of predictions exceeding 1. However, the coefficients of the OLS guarantees almost no predictions above 1.

#### Supplementary Table S14: Predictive performance of candidate models

| Model | MSE | RMSE | MAPE | MPE | R^2^ |
| --- | --- | --- | --- | --- | --- |
| OLS | 0.04354 | 0.20866 | 0.15318 | 2.15 x 10^-14^ | 0.11063 |
| Tobit | 0.04643 | 0.21548 | 0.15524 | -0.04827 | 0.10983 |
| Beta | 0.04446 | 0.21084 | 0.15170 | -0.02266 | 0.10234 |
| GLM: Log-Gaussian | 0.04388 | 0.20947 | 0.15363 | -0.00034 | 0.10377 |
| Two-part GLM: Identity-Gamma | 0.04370 | 0.20905 | 0.15317 | -0.00021 | 0.10740 |
| Two-part GLM: Log-Gamma | 0.04368 | 0.20900 | 0.15320 | -0.00015 | 0.10778 |
| Two-part GLM: Log-Gaussian | 0.04368 | 0.20900 | 0.15324 | -0.00012 | 0.10781 |

MSE: mean squared error; RMSE: root mean squared error; MAPE: mean absolute prediction error; MPE: mean prediction error; R^2^: Efron’s R^2^, square of the correlation between the predicted and observed values.

#### Supplementary Figure S4: Mean prediction error of candidate models in each decile of predicted EQ-5D utility value


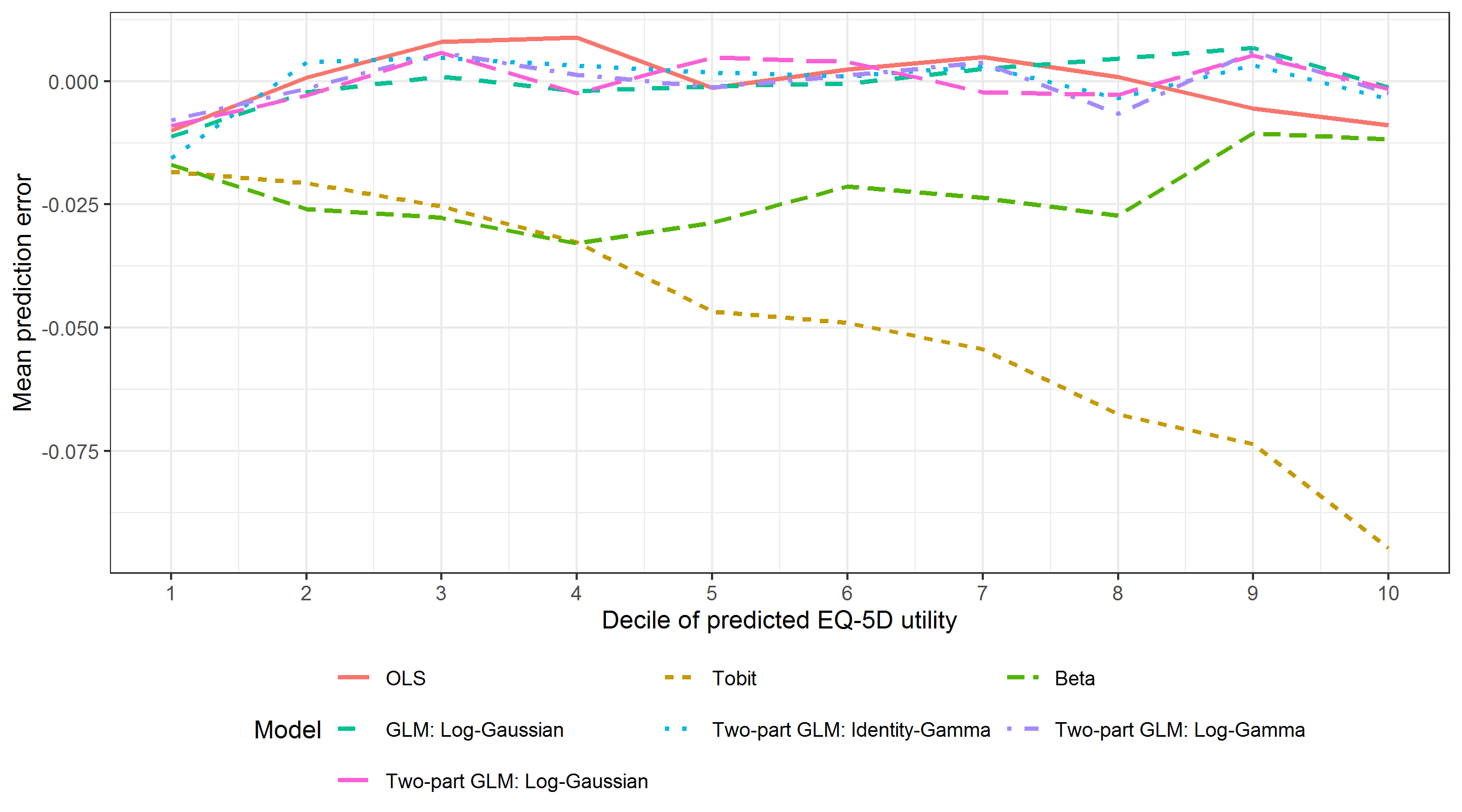


#### Supplementary Table S15: Results from Copas test

|  | Mean (SD) of % | | |  |
| --- | --- | --- | --- | --- |
| Model | 1-𝛼 | 1-𝛿 | 1-𝛾 | % reject |
| OLS | 0 (0) | 3.4 (6) | 3.4 (6) | 18 |
| Tobit | 23.8 (0.5) | 26.3 (4.5) | 3.2 (6) | 100 |
| Beta | 3 (2.1) | 5.9 (6.4) | 2.9 (6.6) | 27 |
| GLM: Log-Gaussian | -1.5 (0.4) | 2.8 (6.5) | 4.3 (6.4) | 16 |
| Two-part GLM: Identity-Gamma | -3.2 (0.7) | -0.6 (6.2) | 2.5 (6.1) | 11 |
| Two-part GLM: Log-Gamma | -1.6 (0.7) | 1.1 (6.2) | 2.7 (6.2) | 12 |
| Two-part GLM: Log-Gaussian | -2.6 (0.5) | 0.4 (6.4) | 2.9 (6.2) | 12 |

𝛼: in-sample calibration - captures effect of mis-specification; 1-𝛼: in-sample shrinkage;

𝛿: out-of-sample calibration - captures both effect of mis-specification and overfitting; 1−𝛿: out-of-sample shrinkage;

1 − 𝛾: combines in-sample and out-of-sample shrinkage - captures effect of overfitting;

% rejected: Percentage of iterations in which Wald test, with null hypothesis that the slope coefficient relating predicted utility to observed utility is 1, is rejected at 5% significance threshold.

#### Supplementary Table S16: Decrements in EQ-5D utility associated with adverse events estimated by the OLS and the two-part GLM

| Disease history (ref: no event) | | Decrements in EQ-5D utility (95% CI) | |
| --- | --- | --- | --- |
|  |  | OLS | Two-part GLM: Identity-Gamma* |
| Transient ischaemic attack | |  |  |
|  | Any history | -0.057 (-0.086, -0.028) | -0.062 (-0.105, -0.024) |
| Ischaemic stroke | |  |  |
|  | Any history | -0.062 (-0.092, -0.032) | -0.065 (-0.132, -0.057) |
| Heart failure | |  |  |
|  | <=1yr | -0.183 (-0.258, -0.108) | -0.195 (-0.137, 0.01) |
|  | >1yr | -0.046 (-0.094, 0.001) | -0.078 (-0.202, -0.095) |
| Non-GI tract cancer | |  |  |
|  | Any history | -0.026 (-0.043, -0.01) | -0.029 (-0.047, 0.002) |
| Intracranial haemorrhage | |  |  |
|  | Any history | -0.164 (-0.251, -0.076) | -0.117 (-0.157, 0.06) |
| GI bleed | |  |  |
|  | Any history | -0.091 (-0.132, -0.051) | -0.094 (-0.114, -0.011) |
| Other major bleed | |  |  |
|  | Any history | -0.096 (-0.146, -0.047) | -0.123 (-0.156, -0.054) |
| Amputation | |  |  |
|  | Any history | -0.206 (-0.259, -0.152) | -0.181 (-0.201, -0.126) |

<=1yr, event occurred within one year prior to EQ-5D questionnaire response; >1yr, event occurred more than a year prior to EQ-5D questionnaire response; GI, gastrointestinal. Other major bleed refers to bleeding events that are not intracranial haemorrhage or GI bleed. Number of participants experiencing each adverse event presented in brackets next to time adverse event occurrence.

*Average marginal effect for two-part model estimated using 1000 bootstrap.^2^

**References**

1. Bilger M, Manning WG. Measuring overfitting in nonlinear models: a new method and an application to health expenditures. *Health Econ*. 2015;24(1):75-85. doi:10.1002/hec.3003

2. Li Z, Mahendra G. Using “Recycled Predictions” for Computing Marginal Effects. In: *SAS Global Forum 2010*. ; 2010.
